# Supplementary figures and images for: Rewiring of master transcription factor cistromes during high-grade serous ovarian cancer development
Source: bioRxiv. 2023 Apr 12:2023.04.11.536378. Preprint. [Version 1] doi: 10.1101/2023.04.11.536378 (PMC10120620; doi:10.1101/2023.04.11.536378)

A)

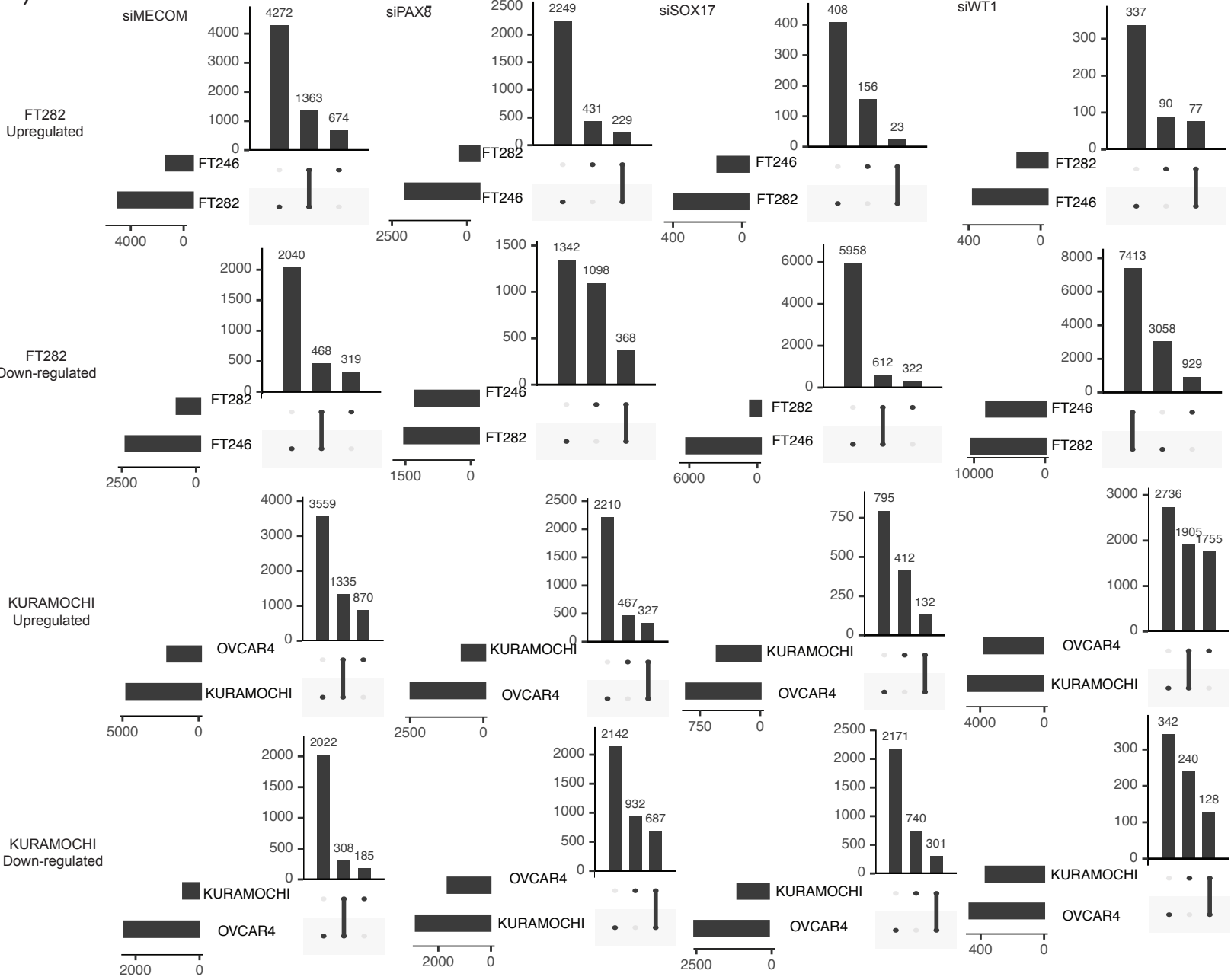

Supplement: Supplement 1 [file NIHPP2023.04.11.536378v1-supplement-1.pdf]
